
JOURNAL INFORMATION
==============================
NLM Title Abbreviation: Aging Cell
Journal ID: Aging Cell
NLM Journal ID: 101130839
Journal ID: ACEL

Aging Cell

ISSN: 1474-9718
EISSN: 1474-9726
Publisher: Wiley

ARTICLE INFORMATION
==============================
PMCID: PMC13384469
DOI: 10.1111/acel.70648
Article ID: ACEL70648
Article version: 1
Subjects: Announcement, Announcement

Anatomical Society Research Studentships 2026/27

Electronic publication date: 2026 Jul 20
Publication date: 2026 Jul
Volume: 25
Issue: 7
Electronic Location ID: e70648
Copyright: © 2026 The Authors. Aging Cell published by Anatomical Society and John Wiley & Sons Ltd.
License: This is an open access article under the terms of the http://creativecommons.org/licenses/by/4.0/ License, which permits use, distribution and reproduction in any medium, provided the original work is properly cited.
License URL: https://creativecommons.org/licenses/by/4.0/

Figure count: 0
Table count: 0
Page count: 3

source-schema-version-number: 2.0
cover-date: July 2026
details-of-publishers-convertor: Converter:WILEY_ML3GV2_TO_JATSPMC version:6.8.4 mode:remove_FC converted:20.07.2026

==============================

### Anatomical Society Research Studentships

### Anatomical Society Research Studentships 2026/27

The Anatomical Society offers a limited number of Research Studentships normally held in University departments whose field of work includes Anatomical Sciences, in the UK and Ireland. Applications are currently being invited from prospective supervisors for Research Studentships to commence in October 2027. The closing date for applications is 5pm Wednesday 19th August 2026.

**For the seventh year**, the Society is reserving a studentship for an **early career investigator (ECI).** An ECI is a person with a lectureship or independent research fellowship who is within 12 years of being awarded a PhD, exclusive of career breaks, who has guaranteed employment for the duration of the studentship and who has not held an AS PhD Studentship previously. The application will be judged according to different criteria than the regular studentship submissions with more emphasis on project design and training provided than on track record. The proposal will still have to meet the high standard expected.

The advertisement, application forms, information and conditions can be found on the website http://www.anatsoc.org.uk/funding-and-awards/grants-and-prizes/anatomical-society-research-studentships. Alternatively, contact the Honorary Secretary, Professor Joanna Matthan, care of maryanne.piggott@kcl.ac.uk

**Closing date for applications is 5pm Wednesday 19th August 2026**

*File: RS ADVERT 202627 Journals v2FINAL post290426Council 070526*
